# Macroscale Static Mechanical Behaviors of Cemented Sand Gravel Dams with Consideration of Construction Interfaces

**DOI:** 10.3390/ma18092068

**Published:** 2025-04-30

**Authors:** Qinghui Liu, Xinzhuo Xie, Long Qian, Xingwen Guo, Xin Cai

**Affiliations:** College of Mechanics and Engineering Science, Hohai University, Nanjing 211100, China; hhulqh@hhu.edu.cn (Q.L.); xxz942845943@163.com (X.X.); 170208020001@hhu.edu.cn (L.Q.); xingwenguo@163.com (X.G.)

**Keywords:** cemented sand and gravel dam, construction interface, finite element method, anti-sliding stability, load-bearing capacity

## Abstract

In order to study the influence of construction interfaces on the safety of middle-low and 100-m cemented sand gravel (CSG) dams, direct shear tests of the construction interfaces with laying mortar and roughening under four different normal pressures are firstly conducted; shear stress–shear displacement curves and interface parameters for the interface models are obtained. Then, finite element models are established using a modified Duncan–Chang constitutive model and a zero-thickness interface model. Displacements, stresses, and anti-sliding stability coefficients of the construction interfaces are obtained, load-bearing capacity is analyzed using the water bulk density overload method, and the obtained results are compared with those of the model without consideration of the construction interfaces. The results show that the obtained displacements and stresses become larger or remain constant when the construction interfaces are considered. The two interface treatment methods (laying mortar and roughening) meet the requirements of anti-sliding stability, and the load-bearing capacity of the construction interface with laying mortar is greater. This study reveals the influence of construction interfaces on the overall mechanical behaviors of the CSG dams and provides technical guidelines for the two construction interface treatments.

## 1. Introduction

The cemented sand and gravel (CSG) dam is a new type of dam whose performance lies between that of the concrete gravity dam and face rockfill dam [1,2,3,4]. Due to its material-saving properties, rapid construction speed, adaptability to soft foundations, excellent seismic performance, economic efficiency, and environmental protection, among others, it is widely used in various hydraulic structures. A CSG dam’s construction begins with foundation preparation, where the ground is leveled, compacted, and reinforced with grouting if necessary. Thereafter, a simple mix of local sand/gravel, cement, and water is blended in a batching plant to form the CSG material. This mixture is placed in 30–50 cm thick layers, with each layer compacted by vibratory rollers to achieve optimal density. Between layers, the surface (construction interface) is roughened or coated with mortar to ensure strong bonding [5,6]. If the construction interface cannot be properly handled, weak interface will be formed between the layers. Therefore, to ensure the safety of the CSG dam, it is crucial to analyze its mechanical performance considering the influence of the construction interface.

Up until now, studies on the mechanical properties of CSG materials have attracted great attention, and various constitutive models, including the linear elastic model [7], equivalent linear elastic model [8], modified Duncan–Chang model [1], improved Saenz’s model [9], elastic–plastic model [10,11,12], damage hypo-plasticity constitutive model [13], and multi-linear ideal softening model [14], have been proposed, laying the foundation for the analysis of CSG dams. The mechanical behaviors of CSG dams were analyzed using a multi-linear ideal softening model based on the finite difference method by Huang and Zhang [14], and the results showed that the failure mode of the dam was plastic yielding. To consider material non-uniformity, the static working behavior of CSG dams was studied by Huang et al. [15] through the elastic random field model. The stress, strain, deformation, and failure mode of the Naheng Reservoir CSG dam were analyzed and discussed using the elastoplastic D-P model in [16]. The Lattice Discrete Particle Model (LDPM) was first used by Chen et al. [17] to model experimental tests of CSG materials, and the behavior of the prototype CSG dam under various loading conditions was also simulated using a multiscale technique in combination with LDPM. The seismic performance of a cemented sand and gravel (CSG) dam was assessed by accounting for two failure modes: tension cracking and base joint sliding [18]. It is noted that the concrete damage plasticity (CDP) model and Mohr–Coulomb friction law were utilized to model the mechanical behaviors of CSG materials and dam–foundation interface, respectively. Based on the improved Saenz’s model, the load-bearing capacity of the CSG dam was evaluated by Zhao et al. [9] using the strength-reduction method and the overload method. A nonlinear finite element program was programmed based on the proposed elastic–plastic model by Wang et al. [10], and numerical simulations of high concrete-faced sand–gravel dams were conducted. Based on the design theories of concrete dams and earth-rock dams, profile design criteria for 100-m CSG dams with different cementing material content under different foundation conditions were proposed by Guo et al. [2]. It is noted that the influence of the construction interface on the global mechanical behaviors of the CSG dams was usually omitted in the above studies.

To incorporate the effect of the construction interfaces of CSG dams, a continuum equivalent model was proposed to simulate the stratified structure of a CSG dam in [8,19], and its stress stability in the static state was analyzed. However, the effect of the construction interface on load-bearing capacity was neglected. For other types of dams, the influence of construction interfaces on their global behaviors was also concerned. The influence of construction interfaces on the dynamic characteristics of roller-compacted concrete (RCC) dams was analyzed by Gu et al. [6], and the results showed that the combination quality of the RCC layers should be ensured to avoid fractures along the construction interfaces. To consider the effect of the construction interface and its seepage, a multipoint hybrid model for RCC arch dam displacement health monitoring was proposed by Liu et al. [20], and the results showed that the displacement field of the dam changed due to the interface.

In summary, although a few studies on the influence of construction interfaces have been explored, their coverage of effects on the global mechanical behaviors of CSG dams are scarce. In this paper, direct shear tests of construction interfaces with roughening and laying mortar under four different normal pressures are first conducted to obtain interface parameters (cohesive stress and friction coefficient) for finite element modeling. Then, a modified Duncan–Zhang model for the CSG material is introduced. Thereafter, finite element models of the middle-low dam and 100-m dam are developed to analyze the effects of construction interfaces on horizontal and vertical displacements, first and third principal stresses, anti-sliding stability, and ultimate load-bearing capacity of the medium-low and 100-m CSG dams after the construction and service periods.

## 2. Direct Shear Test of the Construction Interfaces

### 2.1. Materials

In accordance with the technique guidelines [5], the proper proportions of cement, fly ash, medium-coarse sand, cobblestones, and water were blended to form the CSG material. The cement was P. O. 32.5 ordinary Portland cement produced by Anhui Conch Cement Co., Ltd., Wuhu, China. The fly ash was purchased from a coal-fired power plant in Henan Province, China. The medium-coarse sand and cobblestones from the local markets were selected as the fine and coarse aggregates, respectively. The mass proportions of aggregates with different sizes are shown in Table 1. The mass ratio of cement, fly ash, water, and aggregates was 1:1:2:48.

### 2.2. Specimen Preparation

**Figure 1:**
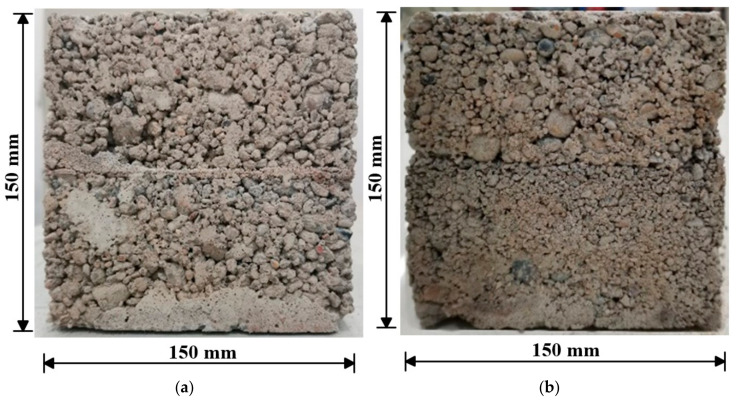
Specimens for direct shear tests: (**a**) laying mortar; (**b**) roughening.

The specimen was designed as a cube with sides of 150 mm and cast in two layers using a special mold. Before casting, the lower sealing plate of the mold was assembled first and coated with an oil-based release agent. Sand, gravel, cement, fly ash, and water were mixed evenly in a mixer. Then, the first layer of CSG was poured into the mold, vibrated by a concrete vibrating table, and compacted. After setting for 24 h, the construction interface was treated. For the interface with laying mortar, cement mortar with a thickness of 3 mm was poured onto the surface of the first layer of CSG. For the interface with roughening, a chisel was used to remove the excess mortar so that coarse aggregates could be seen. Then, a wire brush was used to clean the dust, and the surface of the first layer was wetted for pouring the second layer of the CSG. The upper sealing plate was then assembled, and the upper layer of CSG was poured. After curing for another 48 h under normal temperature conditions, the specimens were demolded and put into the maintenance room (temperature: 25 °C, humidity: 95%) for 90 days of curing. After final trimming and numbering, the specimens shown in Figure 1 were ready for direct shear testing.

### 2.3. Testing

**Figure 2:**
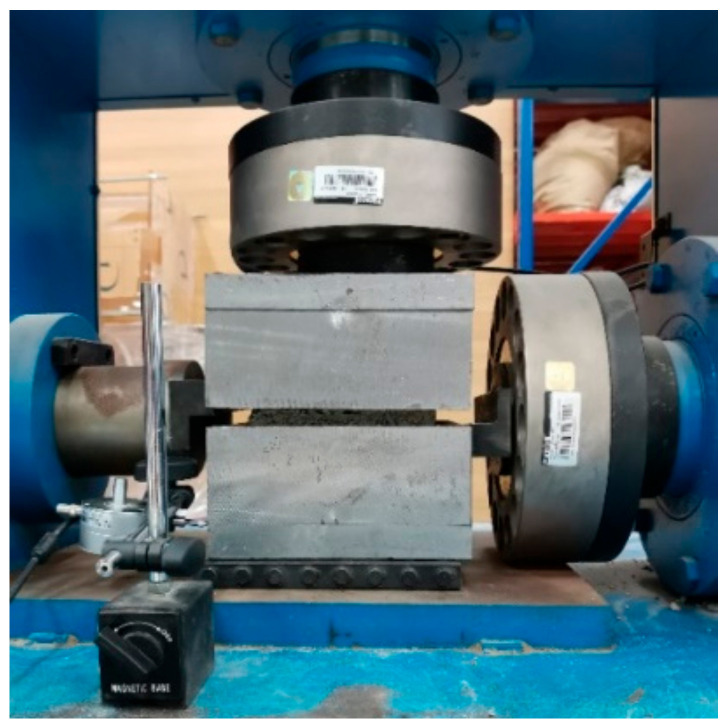
Experimental setup and test configuration.

The tests were performed on a servo-hydraulic testing machine shown in Figure 2, in which the vertical and horizontal loads can be applied simultaneously to measure the shear properties of the CSG materials under different normal pressures. The machine loads and displacements can also be automatically recorded by the testing machine. The shear box for placing the specimen is made of 3 cm-thick steel plates composed of two parts, with internal dimensions of a single shear box being 150 mm × 150 mm × 70 mm. After placing the specimen, a dial gauge is fixed onto the shear box to measure the shear deformation. The normal load (11.25 kN, 22.5 kN, 33.75 kN, and 45.0 kN, corresponding to normal stresses of 0.5 MPa, 1.0 MPa, 1.5 MPa, and 2.0 MPa, respectively) was first applied and kept constant during the test. Subsequently, the shear load was applied under displacement control with a loading rate of 0.36 mm/min until the specimen was damaged.

### 2.4. Test Results and Analysis

**Figure 3:**
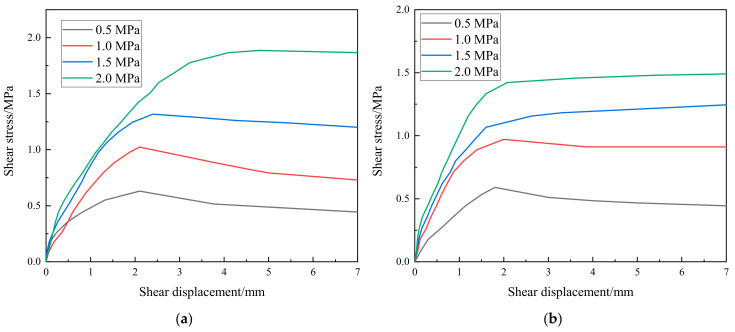
Shear stress–shear displacement curves of the construction interfaces: (**a**) laying mortar; (**b**) roughening.

The shear stress–shear displacement curves for the construction interfaces with laying mortar and roughening are given in Figure 3a and Figure 3b, respectively. It is shown that the shear stress increased rapidly with the shear displacement in the initial loading stage. It attained a peak value and then maintained a relatively stable value as the shear displacement continued to increase for all the loading cases. It was also observed that higher normal pressures led to higher shear stress resistance because higher friction stresses arise at the construction interface due to the normal pressures. Even though slight nonlinear relationships exist between shear stress and shear displacement, and a mild reduction of the shear stress existed for some cases during the last loading stage, the Mohr–Coulomb friction law [18] was used to approximately determine the cohesive stress and friction coefficient of the two types of construction interfaces, and specific values are provided in Table 2. It is seen that the mortar-laid interfaces showed 21.1% higher cohesion and 14.4% higher friction coefficient than the roughened interfaces. It is indicated that the ascending part of the shear stress–shear displacement curve is assumed as a straight line passing through the origin, and the curve corresponding to the last loading stage is assumed as a horizontal straight line.

## 3. Constitutive Model for CSG Material

Due to the nonlinear characteristics of the stress–strain relationship and the low stress level of the CSG dam, a modified Duncan–Zhang model that considers the confining pressure, proposed by Wu et al. [21], is used here. The relationship between strength and confining pressure of the CSG material is given as

(1) $${\left(\sigma_{1}-\sigma_{3}\right)}_{f}=a\cdot p_{a}\cdot e^{b\cdot \frac{\sigma_{3}}{p_{a}}}$$

where *a* and *b* are the fitting parameters, *σ*_1_ and *σ*_3_ are the maximum and minimum principal stresses, respectively, p_a_ is the atmospheric pressure, and the subscript f denotes the state of failure.

The following stress level *S* is introduced:

(2) $$S=\frac{\sigma_{1}-\sigma_{3}}{{\left(\sigma_{1}-\sigma_{3}\right)}_{f}}$$

A cubic function is also introduced to describe the tangential modulus of elasticity *E*_t_:

(3) $$E_{t}=(cS^{3}+dS^{2}+fS+1)\cdot E_{i}$$

where *E*_i_ is the initial tangential modulus of elasticity and *c*, *d*, and *f* are the fitting parameters from the triaxial tests. Substituting Equations (1) and (2) into Equation (3), the initial tangential modulus of elasticity *E*_t_ is obtained as

(4) $$E_{t}=\left[c{\left(\frac{\sigma_{1}-\sigma_{3}}{{\left(\sigma_{1}-\sigma_{3}\right)}_{f}}\right)}^{3}+d{\left(\frac{\sigma_{1}-\sigma_{3}}{{\left(\sigma_{1}-\sigma_{3}\right)}_{f}}\right)}^{2}+f\left(\frac{\sigma_{1}-\sigma_{3}}{{\left(\sigma_{1}-\sigma_{3}\right)}_{f}}\right)+1\right]\cdot E_{i}$$

where *c*, *d*, and *f* are fitting parameters from the triaxial tests and *E*_i_ is the initial tangential modulus of elasticity. The initial tangential modulus of elasticity *E*_i_ is given as

(5) $$E_{i}=k\cdot p_{a}{\left(\frac{\sigma_{3}+p_{a}}{p_{a}}\right)}^{n}$$

where *k* is the exponent of the intercept of the curve lg(*E*_i_/*p*_a_)-lg[(*σ*_3_ + *p*_a_)/*p*_a_] on the longitudinal axis and *n* is the slope of the linear equation, which is determined by the large triaxial compression test.

The Duncan–Zhang principal structure relationship is used to describe the variations of the Poisson’s ratio [1]. Based on the relationship between the Poisson’s ratio of the CSG and confining pressure, the initial Poisson’s ratio *ν*_i_ is expressed as:

(6) $$\nu_{i}=G-F\left(\frac{\sigma_{3}+p_{a}}{p_{a}}\right)$$

where *G* is the Poisson’s ratio when the confining pressure is zero (*σ*_3_ = 0) and *F* is a fitting parameter from the triaxial test. Here, *G* was chosen as 0.223.

By modifying Daniel’s assumption that a tangential Poisson’s ratio changes linearly with the stress level *S*, tangential Poisson’s ratio *ν*_t_ is given as:

(7) $$\nu_{t}=\nu_{i}+(\nu_{\mathrm{tf}}-\nu_{i})S$$

where *ν*_tf_ is Poisson’s ratio when the CSG failed.

Substituting Equation (6) into Equation (7), the Poisson’s ratio for CSG material is obtained as:

(8) $$\nu_{t}=G-F\mathrm{lg}\left(\frac{\sigma_{3}+p_{a}}{p_{a}}\right)+\left[\nu_{\mathrm{tf}}-G+F\mathrm{lg}\left(\frac{\sigma_{3}+p_{a}}{p_{a}}\right)\right]S$$

Here, the parameters shown in Table 3, which were obtained from the triaxial tests, were used. The density of the CSG was chosen as 2320 kg/m^3^.

## 4. Finite Element Models

**Figure 4:**
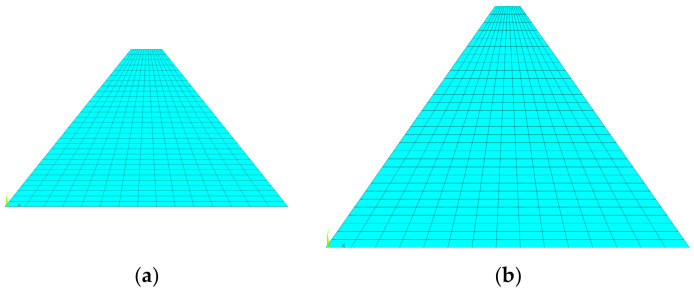
The finite element model: (**a**) middle-low dam; (**b**) 100-m dam.

To describe and analyze the finite element model conveniently, the coordinate system is first clarified here. The origin of the coordinate system is located at the dam heel, and the *x*-axis, *y*-axis, and *z*-axis are along the downstream direction, vertical direction, and the dam axis, respectively. The computational model of the medium-low dam was selected as the Tobetsu Dam in Japan, with a height of 52.4 m, a dam crest width of 8 m, a trapezoidal cross-section with upstream and downstream symmetry, and a slope ratio of 1:0.8. The upstream water depth was 48 m during the impoundment period. The computational model of the 100-m dam was selected as the Oyuk Dam in Turkey. It has a height of 100 m, a crest width of 10 m, a trapezoidal cross-section with upstream and downstream symmetry, and a dam slope ratio of 1:0.7. During the impoundment period, the upstream water depth was 95 m. It was assumed that there was no water downstream, and the water pressure was applied to the upstream concrete face-slab of the dam. The commercial software ANSYS 2020 R2 was used to build the finite element models of the medium-low and 100-m dams. In the models, all the layers were modeled by the 4-node PLANE 182 element with reduced integration and plane strain condition. Secondary developments were conducted to incorporate the modified Duncan–Zhang model presented in Section 3 into the analysis. The construction interfaces were modeled using the Mohr–Coulomb friction law. Zero-thickness contact pairs consisting of Target169 and Contact172 elements were created to model the Mohr–Coulomb friction behaviors of the construction interfaces. The augmented method was used to prevent the mutual penetration of the adjacent layers. The relative sliding between the dam body and dam foundation was neglected. That is to say, all the degrees of freedom of the bottom of the dam were set to zero. The built finite element models for medium-low and 100-m dams are shown in Figure 4a and Figure 4b, respectively.

To simulate the construction process of the dam pouring layer by layer, the element birth and death technique was used. A total of 15 layers were approximately adopted, and the height of each layer was the same, i.e., 14 construction interfaces were uniformly distributed along the height of the dams. The detailed step-by-step procedure is summarized as follows:

(1) Build the overall finite element model of the CSG dam;
(2) Set *i* = 1, kill all the elements except the first construction layer, and solve the finite element model.
(3) Set *i* = *i* + 1, take the calculation results of the last construction layer or loading step as the initial conditions, activate the *i*th layer, and solve the finite element model.
(4) Repeat step (3) until *i* = 15.
(5) Activate the upstream concrete face-slab and apply the water pressure step by step.

## 5. Numerical Results and Discussion

### 5.1. Displacement and Stress

**Figure 5:**
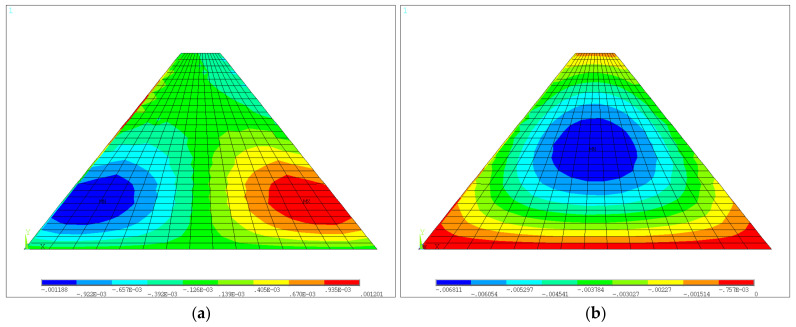
Displacements of the medium-low dam without construction interface after construction: (**a**) horizontal displacement; (**b**) vertical displacement (units: m).

**Figure 6:**
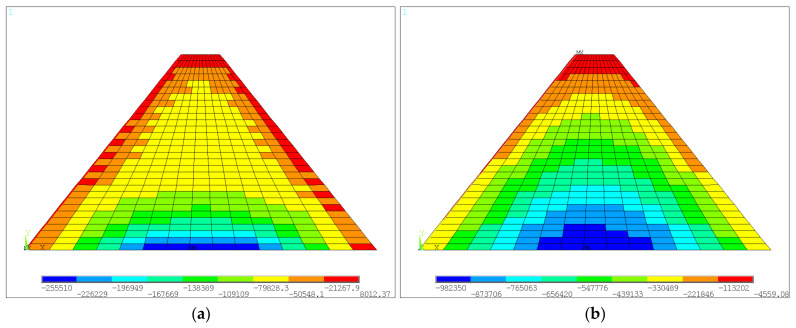
Stresses of the medium-low dam without construction interface after construction: (**a**) the first principal stress; (**b**) the third principal stress (units: Pa).

For the medium-low dam not considering the construction interface after construction, the horizontal and vertical displacement color nephograms are shown in Figure 5a and Figure 5b, respectively. The first and third principal stress color nephograms are shown in Figure 6a and Figure 6b, respectively. It is shown that the maximum horizontal displacements of the upstream and downstream sides of the dam were 1.19 mm and 1.20 mm, respectively, and this displacement was located at about 1/5 of the height of the dam. Due to the consideration of the construction process, the maximum vertical displacement (also called settlement) was 6.81 mm and was located at approximately the middle of the height of the dam. The maximum value of the first principal stress was 0.0080 MPa, which occurred at the dam heel, and the maximum value of the third principal stress was −0.982 MPa, which was located at the axial location of the dam base. The displacements and stress distribution characteristics of the dam with construction interfaces were similar to those of the case without consideration of construction interface. For simplicity, only the maximum values are given in Table 4. It can be seen that larger values of displacements and stresses were predicted when the construction interface is considered. The effects of laying mortar and roughening were almost the same. Specifically, the horizontal displacements showed significant increases (ranging from 90.8% to 105.9%) for the two treatment methods compared to the case of no interface. The vertical displacement had a relatively small increase of 6.0%, the first principal stress had a large increase of 155.0%, and the third principal stress had a small increase of about 3.9%.

**Figure 7:**
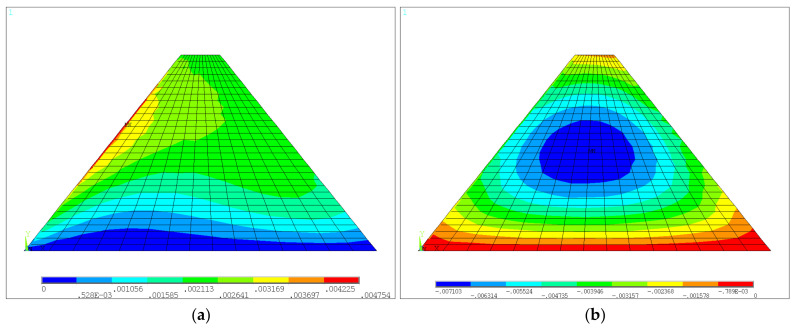
Displacements of the medium-low dam without construction interface after water storage: (**a**) horizontal displacement; (**b**) vertical displacement (units: m).

**Figure 8:**
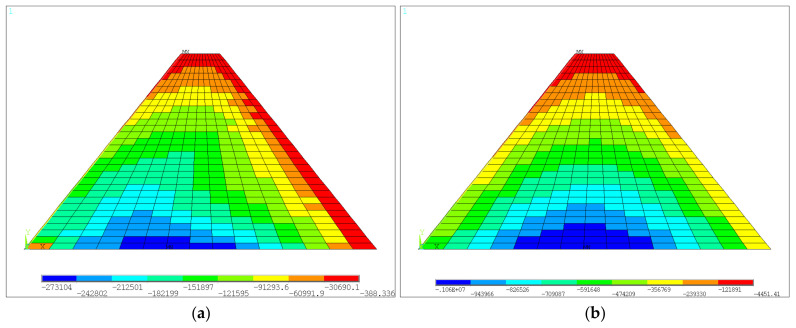
Stresses of the medium-low dam without construction interface after water storage: (**a**) the first principal stress; (**b**) the third principal stress (units: Pa).

For the medium-low dam not considering the construction interface after water was stored, the horizontal and vertical displacement color nephograms are shown in Figure 7a and Figure 7b, respectively. The first and third principal stress color nephograms are shown in Figure 8a and Figure 8b, respectively. It can be seen that the deformation of the dam after water storage was oriented towards the downstream due to the action of upstream water load, and the maximum value increased to 4.75 mm, which occurred in the upstream region shown in Figure 7a. The distribution pattern of the settlement of the dam after the water was stored was similar to that of the dam after construction, and the maximum settlement value was 7.10 mm. The maximum value of the first principal stress was pressure stress, which occurred at the dam heel. The distribution pattern of the third principal stress was also similar to that of the dam after construction, with a maximum value of −1.06 MPa.

The maximum values of deformations and stresses of the medium-low dam with various treatment methods for the construction interface are shown in Table 5. It was observed that larger horizontal displacement (ranging from 98.9% to 99.8%) was predicted when the construction interface was considered, and the vertical displacement increased slightly. The first principal stress showed extremely large increases due to the change from a very small negative value to positive values, and there was no change in the third principal stress. Additionally, the first principal stress of the dam with roughened interfaces was larger than that of the dam with mortar-laying interface because the bonding strength of the construction interface with mortar-laying was larger than that of the construction interface with roughening.

For the 100-m dams, whether with or without construction interfaces, the distribution patterns of deformations and stresses were similar to those of the medium-low dam. The maximum values of deformations and stresses for the 100-m dam after construction are shown in Table 6. For the condition of water storage, the corresponding values are shown in Table 7. It was observed that the presence of construction interfaces increased the displacements and stresses of the 100-m dam, and the impact of the two interface treatment methods on the stresses and displacements was nearly identical. For the condition of the completion period, the maximum horizontal and vertical displacements and the first and third principal stresses were 55.2%, 6.1%, 120.6%, and 5.4% larger than those under the condition without an interface, respectively. For the condition of water storage, the variations of the maximum horizontal and vertical displacements the and first and third principal stresses were 27.2%, 4.0%, 116.5%, and 2.69%, respectively.

### 5.2. Anti-Sliding Stability Analysis

To check the anti-sliding stability of the construction interfaces, the anti-slide safety coefficients of the construction interfaces of the medium-low and 100-m dams were calculated. According to the Technical Guideline for Cemented Granular Material Dams [5], the anti-slide safety coefficient *δ*_kh_ is given as

(9) $$\delta_{\mathrm{kh}}=\frac{\sum_{i=1}^{n}\left(f\sigma_{ni}+c_{i}\right)A_{i}}{\sum_{i=1}^{n}\tau_{ni}A_{i}}$$

where *f* is the friction coefficient of the construction interface, *σ_ni_* and *τ_ni_* are the normal and shear stresses of element *i* at the construction interface, respectively, and *A_i_* is the area of element *i* at the construction interface.

**Figure 9:**
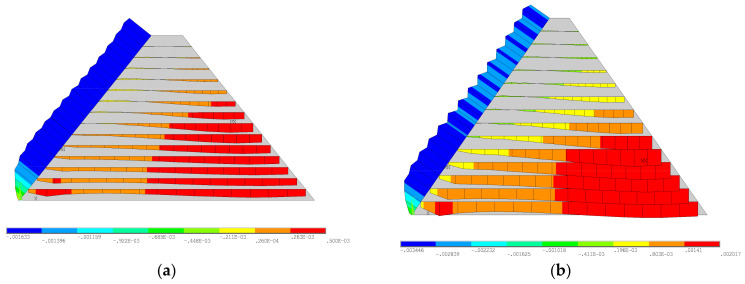
Relative sliding of the construction interfaces: (**a**) middle-low dam; (**b**) 100-m dam (units: m).

The relative sliding of the construction interface for the medium-low and 100-m dams is shown in Figure 9a and Figure 9b, respectively. It can be seen that the maximum sliding occurred at about 1/3 of the dam’s height, which was selected as the control construction interface. The anti-slide safety coefficients for the construction interfaces with laying mortar and toughening are shown in Table 8. It was observed that the anti-slide safety coefficients of the medium-low dam for laying mortar and chiseling were 10.915 and 9.279, respectively. And for the 100-m dam, the corresponding anti-slide safety coefficients were 6.846 and 5.879, respectively. Therefore, the anti-slide safety coefficients were all larger than 3.0, as specified by the technical guideline [5]. Therefore, the two treatment methods of the construction interface were reasonable.

### 5.3. Load-Bearing Capacity Analysis

An overload analysis can evaluate the safety redundancy of CSG dams under increasing water loads. The water bulk density overload method [22] was used here to calculate the load-bearing capacity of the construction interface with maximum sliding. It was assumed that the dam lost its load-bearing capacity when the anti-slide safety coefficient was larger than 3. The anti-slide safety coefficients for construction interfaces with laying mortar and toughening are shown in Table 9. For the medium-low dam, it was seen that when the overload factor was 4, the anti-slide safety coefficient of the construction interface with toughening was less than 3, which was no longer in line with the technical guideline. For the construction interface with laying mortar, when the overload factor was 5, the anti-slide safety coefficient of the construction interface with toughening was less than 3. For the 100-m dam, when the overload factor was 3, the anti-slide safety coefficients of the construction interface with laying mortar and toughening were both less than the value specified by the technical guideline [5].

## 6. Conclusions

In this paper, finite element models of the medium-low and 100-m dams with and without construction interfaces were established, and the influence of interface treatment methods on the deformations and stresses of the dams after construction and water storage were evaluated, and the following conclusions were obtained:

(1) For the medium-low dam after construction, larger values of displacements and stresses were predicted when the construction interfaces were considered, and the increments could reach 105.9% for the horizontal displacement and 155.0% for the first principal stress. The effects of laying mortar and roughening were almost the same.
(2) For the medium-low dam under service condition (water storage), larger values of horizontal displacement (ranging from 98.9% to 99.8%) were obtained when the construction interface was considered, and the first principal stress changed from a very small negative value to positive values. The variations of vertical displacement and third principal stress can be neglected.
(3) For the 100-m dam after construction and water storage, the presence of construction interfaces increased its displacements and stresses, and the impact of two interface treatment methods on the stresses and displacements was nearly identical. For the condition of the completion period (water storage), the variations of maximum horizontal and vertical displacements, and the first and third principal stresses were 55.2% (27.2%), 6.1% (4.0%), 120.6% (116.5%), and 5.4% (2.7%), respectively.
(4) Both treatment methods of laying mortar and roughening were in compliance with the guideline. The anti-slide safety coefficient of the construction interface with laying mortar was usually larger (no less than 15.9%) than that of the construction interface with roughening.

## Figures and Tables

**Table 1:** Mass ratio of aggregates with different sizes.

Aggregate size/mm	0–3	3–5	5–10	10–20
Mass ratio/%	17.0	21.0	27.0	35.0

**Table 2:** Parameters of the construction interfaces.

Treatment Methods	Cohesive Stress *c* (MPa)	Friction Coefficient *f*
Laying mortar	0.345	0.723
Roughening	0.285	0.632

**Table 3:** Parameters of the modified Duncan–Zhang model.

*a*	*b*	*k*	*n*	*c*	*d*	*f*	*G*	*F*
28.13	0.112	11,200	0.2	−0.742	0.476	−0.734	0.23	0.015

**Table 4:** Maximum values of displacements and stresses of the medium-low dam after construction.

Treatment Method	Horizontal Displacement/mm		Vertical Displacement/mm	The First Principal Stress/MPa	The Third Principal Stress/MPa
	Upstream	Downstream			
No interface	1.19	1.20	6.81	0.0080	−0.982
Laying mortar	2.45	2.30	7.22	0.0204	−1.020
Roughening	2.33	2.29	7.22	0.0200	−1.020

**Table 5:** Maximum values of displacements and stresses of the medium-low dam after water storage.

Treatment Method	Horizontal Displacement/mm	Vertical Displacement/mm	The First Principal Stress/MPa	The Third Principal Stress/MPa
No interface	4.75	7.10	−0.00039	−1.06
Laying Mortar	9.49	7.29	0.439	−1.06
Roughening	9.45	7.28	0.601	−1.06

**Table 6:** The maximum value of displacements and stresses of the 100-m dam after construction.

Treatment Method	Horizontal Displacement/mm		Vertical Displacement/mm	The First Principal Stress/MPa	The Third Principal Stress/MPa
	Upstream	Downstream			
No interface	3.35	4.05	19.27	0.0172	−1.660
Mortar	5.20	4.98	20.45	0.0375	−1.750
Roughening	5.20	4.98	20.45	0.0375	−1.750

**Table 7:** The maximum value of displacements and stresses of the 100-m dam after water storage.

Treatment Method	Horizontal Displacement/mm	Vertical Displacement/mm	The First Principal Stress/MPa	The Third Principal Stress/MPa
No interface	19.15	20.46	0.388	−1.860
Mortar	24.36	21.27	0.840	−1.910
Roughening	24.36	21.27	0.840	−1.910

**Table 8:** The anti-slide safety coefficients.

Type of the Dam	Laying Mortar	Toughening
Medium-low dam	10.915	9.279
100-m dam	6.846	5.879

**Table 9:** The anti-slide safety coefficient of the typical construction interfaces.

Overload Factor	Medium-Low Dam		100-m Dam	
	Laying Mortar	Toughening	Laying Mortar	Toughening
1	10.915	9.279	6.846	5.879
2	5.747	4.892	3.676	3.161
3	4.025	3.43	2.619	2.255
4	3.163	2.699	2.091	1.802
5	2.646	2.26	1.774	1.530

## Data Availability

The original contributions presented in the study are included in the article, further inquiries can be directed to the corresponding author.
